# A PAPR Reduction Technique for Fast Touch Sensors Adopting a Multiple Frequency Driving Method on Large Display Panels

**DOI:** 10.3390/s21020429

**Published:** 2021-01-09

**Authors:** Piljoong Kim, Sanghyun Han, Yunho Jung, Seongjoo Lee

**Affiliations:** 1 Department of Information and Communication Engineering and Convergence Engineering for Intelligent Drone, Sejong University, Gunja-dong, Gwangjin-gu, Seoul 05006, Korea; kpj0505@itsoc.sejong.ac.kr; 2 Department Analog and Digital Mixed-Circuit Design and SoC Architecture Design, Leading UI Co., Anyang 14057, Korea; fixfault@leadingui.com; 3 School of Electronics and Information Engineering, Korea Aerospace University, Goyang 10540, Korea; yjung@kau.ac.kr

**Keywords:** capacitive touch sensing system (CTS), large size touch screen, multiple frequency driving method (MFDM), multicarrier transmissions, peak-to-average power ratio (PAPR)

## Abstract

The multiple frequency driving method (MFDM) capacitive touch system (CTS), which drives transmit (TX) electrodes in parallel, has been developed to improve the touch-sensitivity of large touch screens at high speed. However, when driving multiple TX electrodes at the same time, TX signals are merged through the touch panel, which results in increasing the peak-to-average power ratio (PAPR) of combined signals. Due to the high PAPR, the signal is distorted out of the power amplifier’s linear range, causing a touch malfunction. The MFDM CTS can avoid this problem by reducing the drive voltage or partially driving the TX electrodes in parallel. However, these methods cause a significant performance drop with respect to signal-to-noise ratio (SNR) in the MFDM systems. This paper proposes a stack method which reduces PAPR effectively without the performance degradation of MFDM and achieves real-time touch sensitivity in large display panels. The proposed method allocates a suitable phase for each TX electrode to reduce the peak power of combined signals. Instead of investigating all of the phases for the total number of TX electrodes, the optimal phase is estimated from the highest frequency to the lowest one and fixed one by one, which can reduce the required time to find a suitable phase considerably. As a result, it enables high-speed sensing of multi-touch on a large touch screen and effectively reduces PAPR to secure high signal-to-noise-ratio (SNR). Through experiments, it was verified that the proposed method in this paper has an SNR of 39.36 dB, achieving a gain of 19.35 and 5.98 dB compared to the existing touch system method and the algorithm used in the communication system, respectively.

## 1. Introduction

Touch systems are widely used in our society due to the popularization of touch screen technology in user interfaces [1]. The existing touch interface has been applied to the point of sale system (POS), which is used in banks’ unmanned ATMs, and various applications, such as smartphones, tablet PCs, and automobiles that can easily be seen around us. As such, most of the IT devices are equipped with a touch function, which is to be used as an unrivaled user interface, such as electronic boards, augmented reality (AR) touch screens [2], and the smart cars in the future. Moreover, as large displays become popular, the drone control method through the ground control system (GCS) [3] moves from a method that uses devices, such as joysticks to touch devices that use a large display. Since drone control must be performed in real-time, the current touch sensitivity is limited to using touch devices. Therefore, a high-speed touch sensing method is essential for real-time drone control and large-scale touch screens. Various driving methods for these high-speed touch sensing technologies have been studied [4,5,6,7,8,9,10,11,12] in the literature.

**Figure 1:**
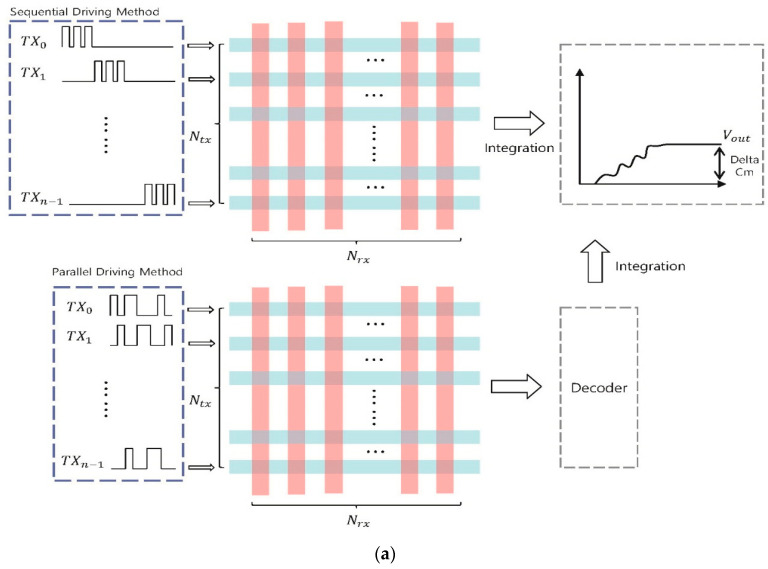

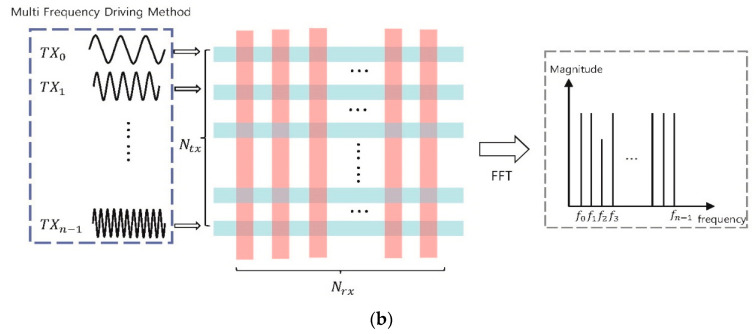
(**a**) Sequential driving method and parallel driving method, (**b**) multiple frequency driving method (MFDM).

Unlike communication systems, a transmit (TX) electrode in the touch system simply generates a pulse signal or a sinusoidal signal without modulated data, and a Rx electrode senses the physical variation of touch nodes for finding the touched point. As shown in Figure 1a, the current touch screen drives a rectangular wave with a constant frequency to each TX electrode sequentially during a specific period, or it gives orthogonality to the TX signals and drives using a parallel driving method. Each method detects the touch point by integrating the received capacitance value and measures the change [4,5,6,7,8]. The sequential driving method [4,5] requires that a certain period of each TX is sequentially driven from TX0 to TXn in order to scan for one frame. Therefore, the larger the screen, the lower the touch sensitivity. The parallel driving method (PDM) scans one frame by driving the TX signal in parallel using orthogonal codes, such as the maximum length [6,7] and the Walsh–Hadamard sequences [8]. Since the TSP is driven in parallel, the SNR is higher than the sequential driving method. When using a small number of TX electrodes, it also can achieve a high frame rate. However, with many TX electrodes, it can cause performance degradation with respect to the frame rate, because the size of orthogonal matrix is proportional to the number of TX electrodes. In order to detect a high-speed touch on many TX electrodes as well as a small number of TX electrodes, each TX electrode is driven simultaneously using a sinusoidal signal with a specific frequency, which is shown in Figure 1b. With this method, a multiple frequency driving method (MFDM) as a frequency domain signal processing method [9,10,11,12] has been developed that scans one frame by driving all of touch nodes at once.

### 1.1. System Overview of MFDM

**Figure 2:**
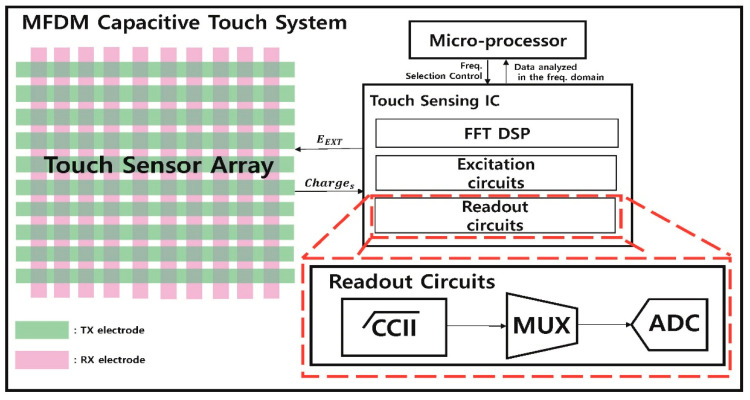
Block diagram of MFDM CTS structure [11].

Figure 2 is a block diagram of an MFDM CTS [11], which consists of the microcontroller unit (MCU), the touch screen panel (TSP), and the touch sensing integrated circuit (IC), which includes the excitation circuits, the readout circuit, and the fast Fourier transform (FFT) processor. The MFDM system measures the external noise and selects a frequency of signal for each TX electrodes with low noise. The MCU performs the FFT pre-processing to measure the external noise in order improve the SNR. Additionally, during driving, the MCU continues to measure the noise level of frequencies allocated to the TX electrode. When the noise above the threshold is measured, the TX electrode is reassigned to the frequency with low noise. When the FFT pre-processing is advanced, the touch sensing IC does not emit the $E_{EXT}$ to the TSP and sends the $D_{FFT},$ which is the FFT data to the MCU at the FFT processor. The value of the $D_{FFT}$ changes because of the process variation of the TSP and the channel mismatch. It is compensated by using a finite impulse response (FIR) filter and correlated double sampling (CDS) method, so the external noise is minimized, and the frequency is selected using the algorithm from [11]. With the frequency received from the MCU, the excitation circuits of the touch sensing IC generate the signal $E_{EXT,}$ which is shown in Equation (1), and it emits the $E_{EXT}$ to the TX electrode of the TSP.

(1) $$E_{EXT}=\cos(2\pi \frac{f_{i}}{L}t)$$

where $f_{i}$ is the FFT bin of the frequency selected by the MCU, L is the FFT length, and t is the sampling time. The $E_{EXT}$ emitted in parallel for each TX electrode passes through the touch panel, and it is combined and sent to the readout circuits as a charge signal, which is the $Charge_{s}$ through the RX electrode. After passing the readout circuits, the amplified $Charge_{s}$ completes the FFT, and it is sent to the MCU to obtain touch information that has touch points. The touch point’s capacitance value decreases when touched, and the FFT magnitude also decreases, so the touch point can be found even if it is driven in parallel. These MFDMs can have higher touch sensitivity and SNR as the number of TX electrodes driven simultaneously increases. However, if the $Charge_{s}$ has a high PAPR [13], the signal is distorted out of the linear range of the power amplifier, which results in an error in the touch sensing. In the conventional [9,10,11], in order to reduce the PAPR, a Charge Overflow Protection Block (COPB) was added to the Current Conveyor II (CCII) [11] of the readout circuits, and the input impedance was converted to 4-bit control data (D [3: 0]) in order to reduce the voltage (drive voltage) of the input signal ($Charge_{s}$). However, when the driving voltage is lowered, the SNR decreases, so the noise immunity is weakened. There is a limit to the amount of increase in the touch screen size because the more the TX signal is used, the lower the driving voltage is. Additionally, [12] multiplies the TX signal by a sinusoid signal having a frequency of $f_{d}$/2 to shift the frequency of signal for offsetting the signal one another. After that, the signal is restored via the demodulation process (reverse work) in the RX stage, and the touch point can be found. However, when the amplitude of the signal becomes 0 by offsetting process, the original signal can be distorted in the demodulation process, and the complexity of the touch sensing IC also increases. The proposed method in this paper improves the SNR compared to the conventional method by solving the charge overflow without using the COPB of the CCII, which reduces the driving voltage, and the demodulation process.

### 1.2. The PAPR Reduction Method in the Communications System

**Figure 3:**
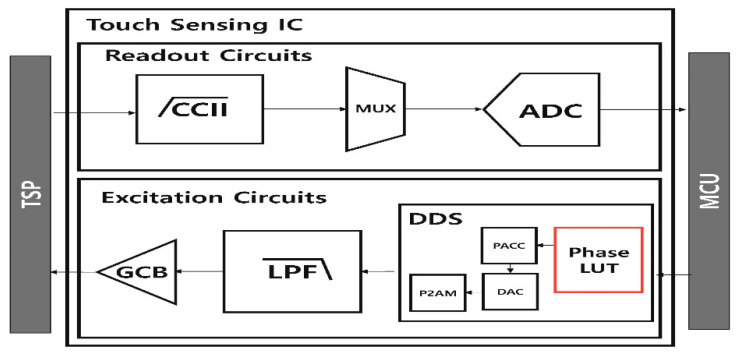
Block diagram of the P-PTS that is used for the communications system.

**Figure 4:**
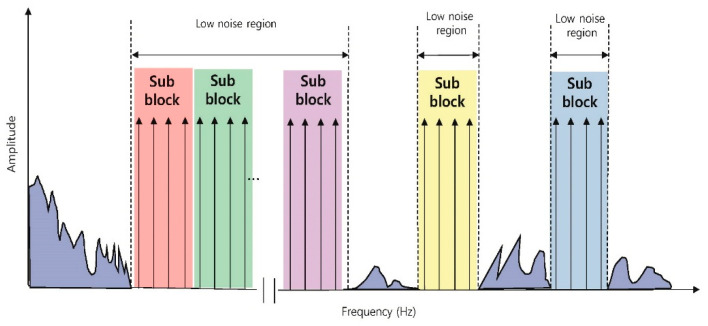
P-PTS selects frequencies in subblock units, which have 4 TX signals.

The PAPR problem of the MFDM also occurs in multicarrier transmissions [13,14,15,16,17,18,19,20,21,22,23], such as orthogonal frequency division multiplexing (OFDM) [13], discrete multi-tone (DMT) [14], and universal filtered multi carrier (UFMC) [15,16] in the communications system. In the communications system, various PAPR reduction studies have been conducted. Among them, the representative techniques in order to reduce the PAPR without distorting the signal are the coding technique [17,18], selective mapping (SLM) [19,20], and partial transmit sequence (PTS) [21,22,23]. Each method shifts the phase of the input data in order to minimize the PAPR. It will compare how much phase should be shifted for each input data to have the minimum PAPR. The coding technique searches for the optimal phase codewords that minimize the PAPR for the entire input data and applies those codewords. The SLM makes some candidate phase codewords and applies each codeword to the input data. The SLM then selects the codewords that generate the minimum PAPR. Regarding the PTS, there are too many cases to find the optimal phase of the entire input data every time, so the input data is divided into several sub-blocks in order to find the optimal phase for each sub-block to reduce the PAPR. In an MFDM system that uses a different frequency for each driving and uses many TX electrodes, it is impossible to find the codewords and the optimal phase each driving due to many computations. Additionally, utilization of candidate codewords causes a malfunction in touch detection due to irregular and insufficient PAPR reduction. For these reasons, the PAPR reduction techniques that is used in the communications system cannot be directly applied to the MFDM CTS. However, when adding a LUT to the DDS of the MFDM CTS like Figure 3, the PTS in the communication system can be applied to the MFDM CTS. In this paper, such a method is referred to as P-PTS. Similar to the PTS, the P-PTS divides the frequency band into sub-blocks by a block slicer and compares PAPR within the sub-block. Each sub-block is multiplied by the phase factor to compare the PAPR, and the phase factor that has the minimum PAPR is stored in the LUT. When a frequency is selected in the MCU, the phase factor that corresponds to that frequency is taken from the LUT and the $E_{EXT}$ is generated, so the PTS with a large amount of computation can be applied to the MFDM CTS. However, since the MCU selects frequencies in the sub-block units, which is shown in Figure 4, it is more susceptible to external noise than the conventional MFDMs, which select frequencies one by one. Additionally, there is a drawback in the P-PTS that the PAPR reduction effect cannot be seen when selecting the sub-blocks that have the same phase.

This paper proposes the stack method, which enables high-speed touch sensing by reducing the PAPR effectively with a small number of computations without degrading the performance of the MFDM. The remainder of this paper is organized as follows. Section 2 describes the Stack, which is the PAPR reduction algorithm proposed in this paper. In Section 3, the PAPR reduction algorithm in Section 1 and the proposed algorithm in this paper are applied to compare the amount of the PAPR reduction. The improvement of the SNR for the PAPR reduction is verified through experiments. Section 4 summarizes the results and concludes this paper.

## 2. The PAPR Reduction Method about Stack

On account of the large display’s popularization, the demand for large touch screens increases in various fields, such as GCS, electronic blackboards, and smart cars. There is presently a limit to the applicability of touch systems to large touch screens, so the MFDM was developed for high-speed sensing. However, it is difficult to use many TX electrodes because of the high PAPR problem of the MFDM. Therefore, the PAPR reduction is indispensable to increase the number of TX electrodes for high-speed touch sensing. The PAPR reduction algorithm in a multicarrier transmission system, such as the communications systems cannot be applied to a large touch screen using a large amount of TX electrodes due to the number of calculations. Without testing all of the phases of the TX electrodes at the same time, the proposed stack method finds sequentially the optimal phases of the TX electrodes with from the highest frequency to the lowest one. In the proposed method, the optimal phase of *i*-th TX electrode can be obtained and set with only considering the phases of *i*+1 to (*N*-1)-th TX ones (the frequency of *i*-th TX electrode is lower than *i*+1-th TX one). Since the PAPR of the TX electrodes is not compared at the same time, not only the phase coefficient is detected at high speed, but real-time touch detection is possible even when many TX electrodes are adopted to an MFDM system in which the frequency changes with every driving.

**Figure 5:**
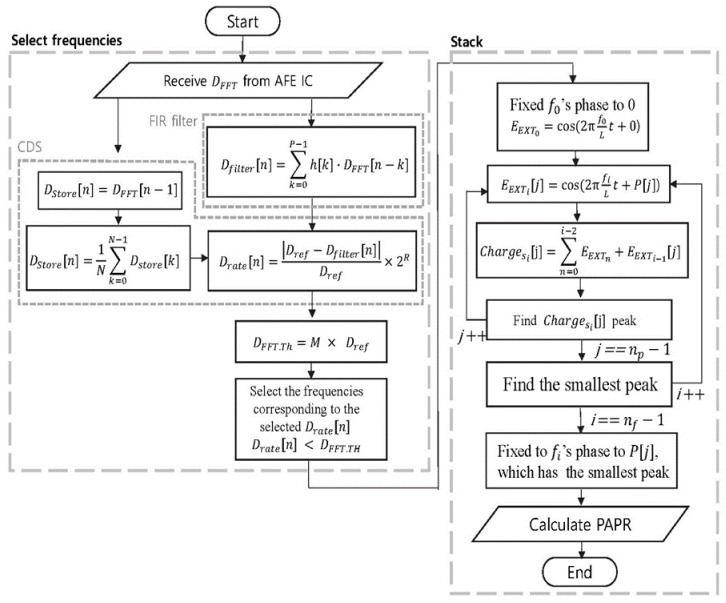
The MFDM algorithm [11] with stack.

**Figure 6:**
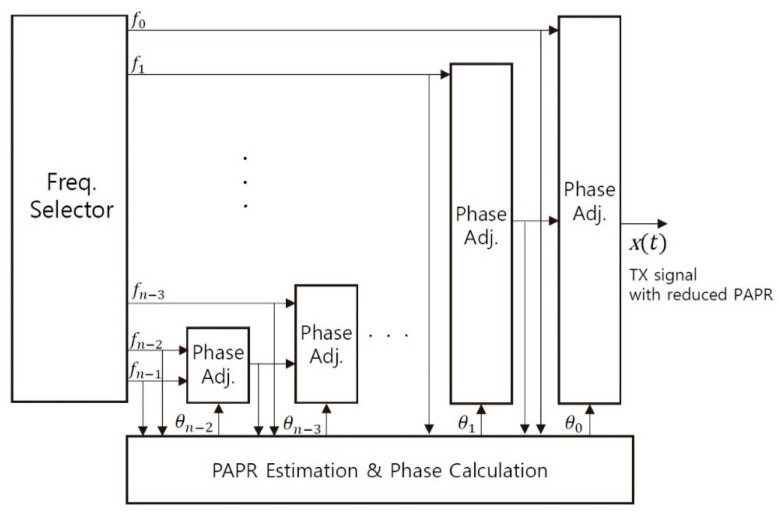
Block diagram of the proposed stack method in the MFDM system.

Figure 5 and Figure 6 show the algorithm and the block diagram of the proposed stack method in the MFDM system, respectively. The MCU receives the $D_{FFT}$ from the FFT processor of the touch sensing IC, and it selects the frequency in the region with less external noise through the FIR filter and the CDS. Whenever the external noise exceeds the pre-determined threshold even during driving, the proposed method performs the process finding re-allocable frequency and its optimum phase for each TX electrode in the background without affecting the current touch sensing process. The proposed method has about the latency of tens of milliseconds to decide the frequency and phase candidates for the TX electrodes at the clock speed of 100 MHz, which is just the experimental environment of this paper and can be reduced depending on the clock speed. However, since the re-assignment and re-generation process for the TX electrodes takes less than about 1 ms and the touch response time requirement is about 8 ms (120 Hz), the proposed algorithm in the MFDM system does not affect the normal touch sensing process. The selected frequencies are sorted in the order of the lower band frequency and the phase then is allocated. The high-band frequency provides many changes in the signal overlapping even if a little phase shift occurs, but the low-band frequency does not significantly affect the PAPR reduction by the phase shift compared with the high-frequency band. That is why the phase in Equation (2) is assigned from the highest band frequency.

(2) $$P\left[j\right]=\frac{2\pi }{n_{p}}\times j\;\left(0\leq j<n_{p}\right)$$

Equation (2) means an array P, which 2π is divided by the number of phases $n_{p}$. Among the rearranged frequencies, the first frequency $f_{0}$ is fixed to $0^{\text{°}}$. After that, the $f_{1}$ frequency generates the $E_{EXT1}\left[0\right]$ by shifting the $E_{EXT}$ of Equation (1) by P[0], which is the first phase stored in the array P. This way, the signal $E_{EXT1}$($E_{EXT1}\left[0\right],\;E_{EXT1}\left[1\right],\;\cdots ,E_{EXT1}\left[n_{p-1}\right]$) is generated using P($P\left[0\right],\;P\left[1\right],\cdots ,P\left[n_{p}-1\right]$) and $f_{1}$. A total of $n_{p}$ number of $E_{EXT1}$ are created, and $Charge_{s1}$($Charge_{s1}$ [0], $Charge_{s1}$ [1],⋯, $Charge_{s1}$[$n_{p-1}$]) is generated by adding the $E_{EXT1}$ to the signal $E_{EXT0}$ that is generated using $f_{0}$ fixed at $0^{\text{°}},$ which is shown in Equation (3).

(3) $$Charge_{s_{i}}\left[j\right]=\overset{i-2}{\underset{n=0}{\sum }}E_{EXT_{n}}+E_{EXT_{i-1}}\left[j\right]\;\left(0\leq j<n_{p}\right)$$

(4) $$\mathrm{PAPR}=\;\frac{Peak^{2}}{\frac{\sum_{i=0}^{t-1}Charge_{s}{\left[i\right]}^{2}}{t}}$$

From Equation (4), the PAPR is proportional to the square of the signal’s peak. Accordingly, the peak value of each of the generated number of phases $n_{p}$ $Charge_{s1}$ signals is found. Accordingly, the peak value of each of the generated $n_{p}$ number of $Charge_{s1}$ signals is found. $Charge_{s1}\left[j\right],$ which produces the smallest peak and fixes the phase that creates $Charge_{s1}\left[j\right],$ is found at that time to the phase of $f_{1}$. This process is repeated for $n_{f}$ number of frequencies selected by the MCU, and the phase is fixed for each frequency. Using this method, the phase that produces the smallest peak is fixed to that frequency while stacking the frequencies one by one from the slow frequency to the high frequency. The PAPR reduction effect is excellent, because the phase that generates the minimum peak is selected while adding the $E_{EXT}$ signals generated at the frequency selected from the MCU one by one. Moreover, since the PAPR of all of the TX electrodes is not compared at the same time, the PAPR comparison calculation amount does not increase exponentially with the numbers $n_{TX}$ and $n_{p}$ of the TX electrodes. Equation (5) is the PAPR comparison calculation amount of the Stack, which is significantly smaller than the P-PTS calculation amount. When the stack method is applied to the MFDM system, it can find phase to reduce the PAPR effectively in real-time.

(5) $$n_{PAPR\_STACK}=\left(n_{TX}-1\right)*n_{p}$$

**Figure 7:**
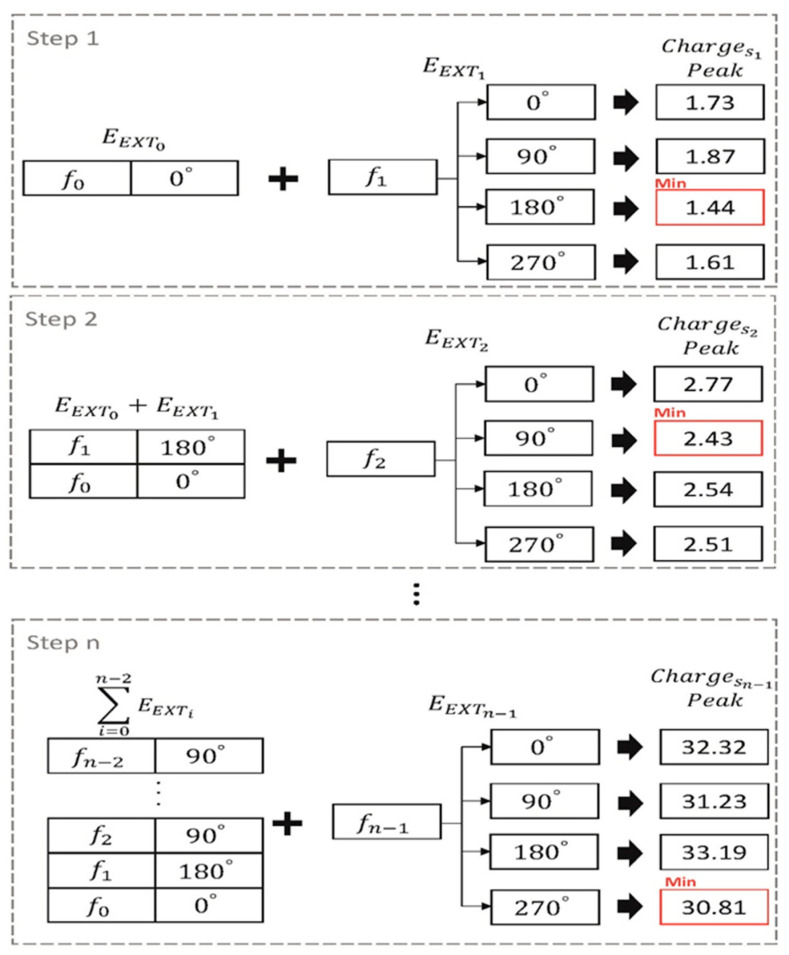
Example of the proposed algorithm using *n* TX electrodes and $n_{p}=4$ ($0^{\text{°}}$, $90^{\text{°}}$, $180^{\text{°}}$, $270^{\text{°}}$).

Figure 7 shows an example of a Stack that finds the phase of the TX electrode frequency using n number of TX electrodes and four phases, which include $0^{\text{°}}$, $90^{\text{°}}$, $180^{\text{°}}$, and $270^{\text{°}}$. In Step 1, add $E_{EXT0},$ which is phase shifted by $0^{\text{°}},$ and the signal $E_{EXT1}$ generated by $\;f_{1}$ and phase $0^{\text{°}},\;90^{\text{°}},\;180^{\text{°}},\;\mathrm{and}\;270^{\text{°}}$, respectively. After that, the value of $Charge_{s1}$ made by combining $E_{EXT0}$ and $E_{EXT1}$ is compared. The $Charge_{s1}$ has the smallest value of 1.44 when moved $180^{\text{°}}$, and $180^{\text{°}}$ is assigned to $\;f_{1}$’s phase. Step 2 repeats the process of Step 1 using the $E_{EXT0}$ and the $E_{EXT1}$ emitted in the previous step. Using this method, it is repeated n times by the number of TX electrodes in order to search for a combination of frequency and phases.

## 3. Experimental Result 

The P-PTS and the stack were applied to the experimental environment in Table 1 in order to calculate and compare the PAPR values. Coupling occurs in the low band frequency, and the high band frequency is noisy, so the frequency in the band from 150 to 1 MHz is used. The number of usable frequencies is 288 when considering the 3 MHz ADC and the 1024 FFT points, and the PAPR was calculated when 64 TX electrodes were used. In the stack case, the operating speed was measured at a 100 MHz clock on the zynq-7020 board to verify that the optimum phase was found for real-time. The P-PTS divides 288 frequencies into 144, 64, and 36 sub-blocks by grouping the $sb_{TX}$ into 2, 4, and 8 number of TX electrodes per sub-block. The P-PTS divides 288 frequencies into $sb_{TX}$ (2, 4, 8) to create 144, 64, and 36 sub-blocks, respectively. The $sb_{TX}$ means the number of TX electrodes for each sub-block. In order to allocate the frequencies to 64 TX electrodes, in the case of the P-PTS 2 (P-PTS when $sb_{TX}$ is 2), 32 sub-blocks should be selected out of 144 sub-blocks. When selecting 32 sub-blocks out of 144 sub-blocks, it is impossible to complete the enumeration for about $10^{32}$ signal candidates for the PAPR comparison. Similarly, in the P-PTS 4 and the P-PTS 8, too many signal candidates could not be enumerated entirely, so 10 million simulations were performed. The maximum PAPR and the average PAPR were calculated during the 10 million simulations. At this time, the PAPR does not decrease when the sub-blocks, which all have the same phase, are selected, so this case was excluded and measured. Similar to the P-PTS, the stack cannot perform a complete the enumeration, so it was simulated 10 million times. Using the 10 million simulations results, the touch panel was modeled using a MATLAB Simulink and the PAPR reduction and the touch points were verified. Additionally, the 42-inch MFDM CTS was implemented and verified using real-time sensing.

**Figure 8:**
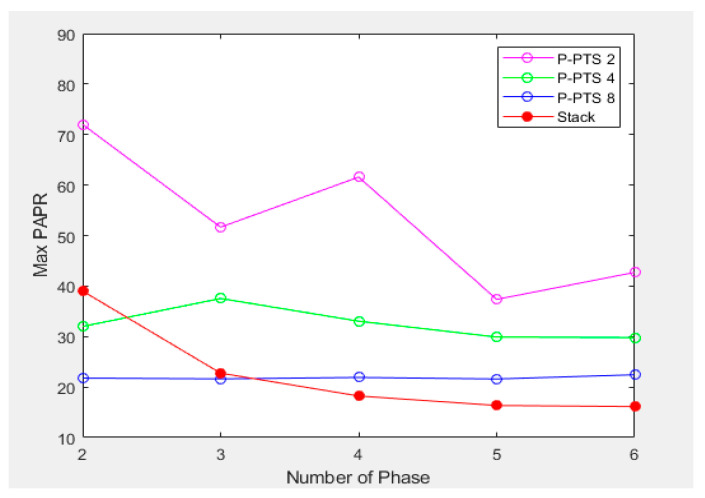
P-PTS and stack’s maximum PAPR when using 2, 3, 4, 5, and 6 phases.

Figure 8 is a graph that compares the maximum PAPR of 10 million simulations of the P-PTS and the Stack. The maximum PAPR was measured for each case by applying 2($0^{\text{°}}$, $180^{\text{°}}$), 3($0^{\text{°}}$, $120^{\text{°}}$, $240^{\text{°}}$), 4($0^{\text{°}}$, $90^{\text{°}}$, $180^{\text{°}}$, $270^{\text{°}}$), 5($0^{\text{°}}$, $72^{\text{°}}$, $144^{\text{°}}$, $216^{\text{°}}$, $288^{\text{°}}$), 6($0^{\text{°}}$, $60^{\text{°}}$, $120^{\text{°}}$, $180^{\text{°}}$, $240^{\text{°}}$, $300^{\text{°}}$) $n_{p}$. The P-PTS 2 has an unstable maximum PAPR based on the $n_{p}$, but the P-PTS 4 and the P-PTS 8 show a tendency that the maximum PAPR decreases as the $n_{p}$ increases. However, in the P-PTS 4, the amount of the PAPR reduction is small from the $n_{p}$ of 6, and in the case of the P-PTS 8, the amount of the PAPR reduction according to the $n_{p}$ is insufficient. Therefore, increasing the $sb_{TX}$ rather than increasing the $n_{p}$ is effective to reduce the PAPR in P-PTS. Additionally, it has a more stable maximum PAPR as the $sb_{TX}$ increases. However, the MFDM CTS selects the frequency of the sub-block units with less external noise, which is shown in Figure 5. As $the\;sb_{TX}$ increases, the sub-block becomes more massive and is more affected by the external noise, so the SNRs performance drops.

The stack has a higher PAPR than P-PTS 4 and P-PTS 8 when two phases are used, but it has a lower PAPR than P-PTS even when four phases are used. Moreover, as $n_{p}$ is increased, the maximum PAPR ($PAPR_{m}$) and the average PAPR ($PAPR_{a}$) decrease, which shows a better performance than the P-PTS. Table 2 shows the results of the Stack measuring the maximum PAPR, the average PAPR, and the operating time (t) by applying up to 128 $n_{p}$. $PAPR_{a}$ and $PAPR_{m}$ decrease proportional to $n_{p}$, while t increases with $n_{p}$. Therefore, in order to find the effective value of $n_{p}$, a PAPR-time performance (*PAPR*-*T*) is defined by

(6) $$PAPR-T=PAPR_{a}*\;PAPR_{m}*t$$

In the evaluation for various $n_{p}$, the value of $n_{p}$ is selected with which a PAPR-time performance (*PAPR*-*T*) does not change abruptly.

Table 3 compares the computational complexities of the P-PTS and stack methods. The computational complexity means that how many times compare operations are need for finding the optimal phase in each algorithm. The stack method can find the optimal phase 9 times faster than P-PTS. In addition to that, both the maximum and the average values of PAPR also be reduced by about 40% than P-PTS.

**Figure 9:**
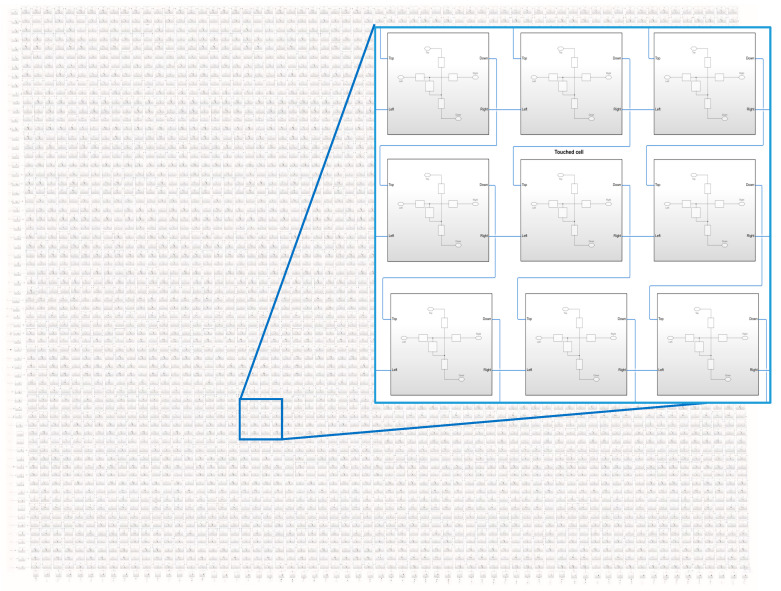
64 × 64 capacitive touch screen panel implemented using MATLAB Simulink.

As shown in Figure 9, the algorithm was verified with a 64 × 64 capacitive touch screen panel model using MATLAB and Simulink, which reflected the characteristics of the panel’s actual passive elements. The resistance was set to 2.5Ω. The capacitance of the untouched and touched panel were set to 1.5 and 1.2 pF, respectively. The maximum number of multi-touch points was 10. Considering the experimental environment, external noise, sensing, and PAPR reduction, the P-PTS 8 phase 2 and the stack phase 16 is suitable for the MFDM CTS. Therefore, the combination of the frequency and the phase was applied when the maximum PAPR of the P-PTS 8 phase 2 and the stack phase 16 occurred. The improved SNR was measured by amplifying the average power as much as the reduced PAPR in each method. The experiment verified the five multi-touch point touch sensing, which assumed that the touch was made by reducing the five touch panels’ capacitances.

**Figure 10:**
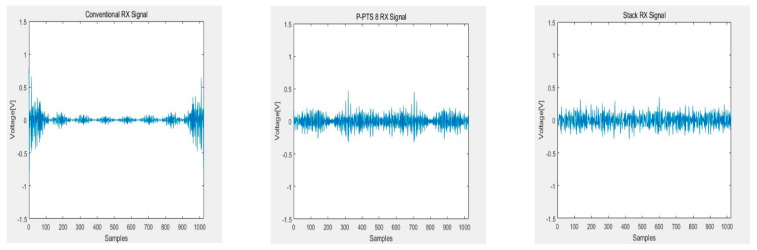
RX signals without amplifying average power.

**Figure 11:**
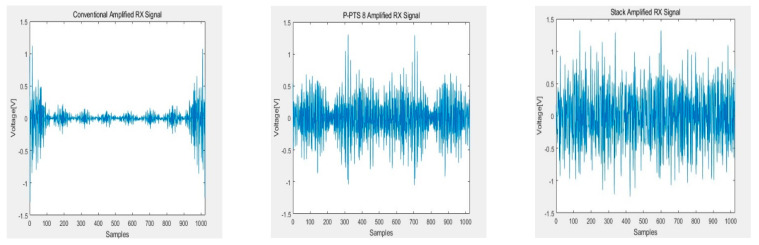
RX signal with an amplified average power that was not to exceed the linear range of the power amplifier.

**Figure 12:**
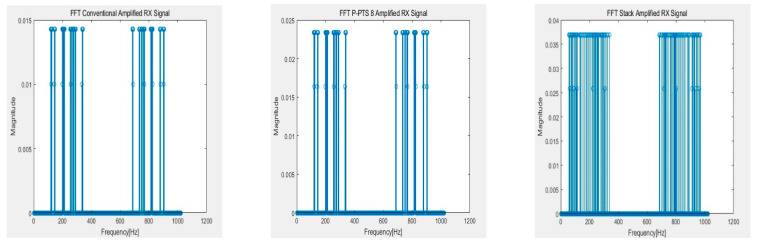
Fast Fourier transform (FFT) of RX signal with amplified an average power that was not to exceed the linear range of the power amplifier.

**Figure 13:**
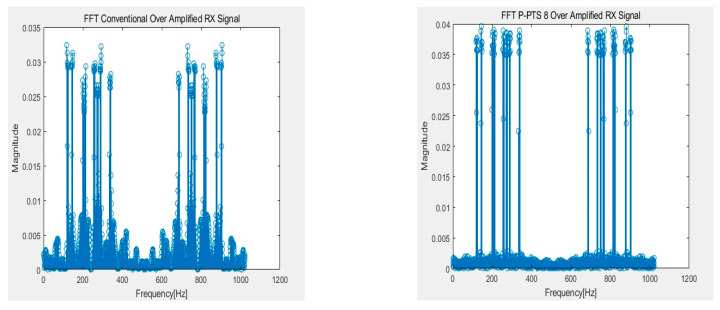
Hazard on the FFT of the conventional RX signal and partial transmit sequence (PTS) RX signal when amplified equally as Stack.

Figure 10, Figure 11, Figure 12 and Figure 13 show the RX signals and the RX signals FFT when applying a combination of the frequency and the phase when the maximum PAPR of the P-PTS 8 phase 2, and the stack phase 16 occurs on the 64 × 64 capacitive touch screen panel, which is shpwn in Figure 9. The RX signals indicated the signal magnitude from −1.32 V to 1.32 V according to the power amplifier’s output range. Figure 10 shows the RX signal with no average power. These are the RX signals where the conventional, the PTS Block 8 phase 2, and the stack phase 16 are applied, respectively. In the conventional method, the value of 0 for the RX signal has a high peak of 0.7851. The peaks of the P-PTS and the stack are 0.465 and 0.3534, and the stack has the lowest peak. The stack peak is 54.99% lower than the conventional. Figure 11 shows that the peak values of the RX signals in Figure 10 were amplified, so it does not exceed the linear range of the power amplifier. The stack has more PAPR reduction than the conventional and the P-PTS, so a lot of amplification is possible. The stack has a high SNR at 39.36 dB. On the other hand, the conventional and the PTS have low SNRs, which are 20.01 and 33.38 dB, compared to the stack. Figure 12 confirms in the frequency domain that the signal distortion does not occur even if the signal is amplified by applying the stack. It shows that all five multi-touch are sensed correctly. Figure 13 shows the FFT when the conventional RX signal and the P-PTS RX signal’s average power are amplified the same as the stack. In both cases, the RX signal exceeds the power amplifier’s linear range, which causes signal distortion. It causes harmonics in the frequency domain, which makes it difficult to determine the touch point. Additionally, a touch malfunction can occur with only a small amount of noise.

**Figure 14:**
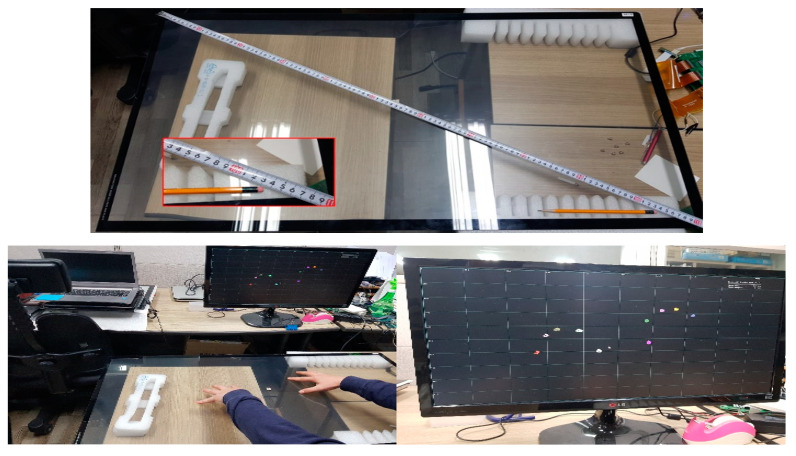
Experimental environment for real-time sensing of 10 multi-touch on a 42-inch MFDM capacitive touch system (CTS) and output to a monitor.

**Figure 15:**
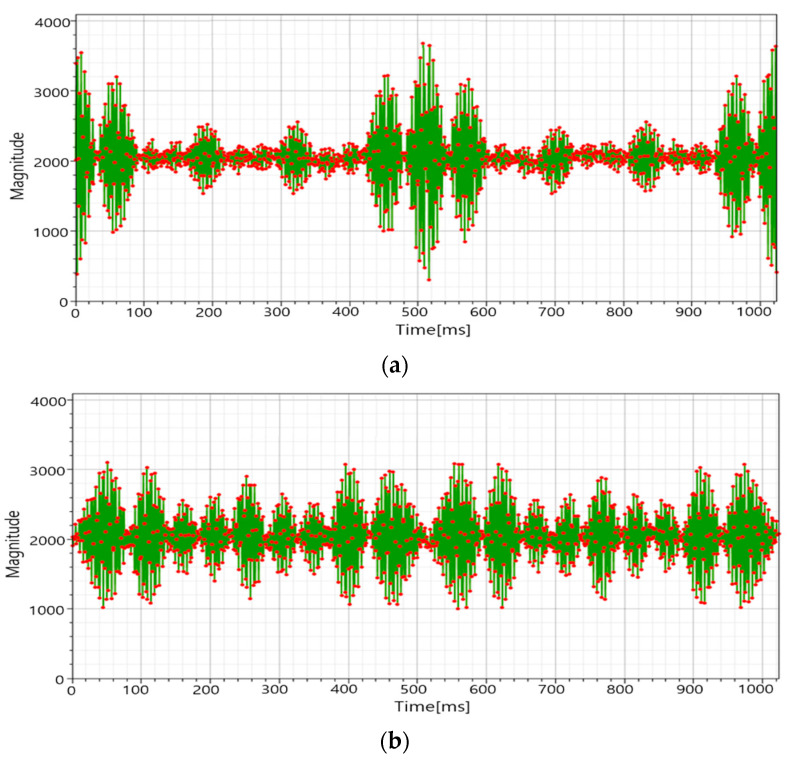
(**a**) The conventional CTS sequential driving method, and (**b**) the concurrent driving method.

Figure 14 shows the proposed algorithm test environment on an actual 42-inch touch panel. When ten touches were made on the touch panel, the touch panel’s multi-touch points were outputted to the monitor in real-time, and it was verified that the touch points were correctly detected. Figure 15 shows the ADC data of the RX signal when it was experimented in the experimental environment of Figure 14. Figure 15a is the ADC data of the RX signal generated when the conventional MFDM CTS is touched, and Figure 15b is the ADC data of the RX signal verified by applying the provided stack phase 2. From the results of Figure 15b, it was verified that the peak decreased when the proposed method was used in the actual MFDM CTS. The touch point at this time was outputted to the monitor in real-time and verified.

## 4. Conclusions

The PAPR reduction method that was used in the existing MFDM CTS lowers the driving voltage or partially drives the TX electrodes in parallel, which thereby degrades the MFDM performance. In addition, the PAPR reduction method used in the communications systems cannot perform real-time sensing due to a large number of computations when applied to the MFDM CTS with variable frequency. This paper proposed a real-time touch sensitive method that reduces the high PAPR that occurs when the MFDM, which is a frequency domain signal processing method, is applied to the CTS without degrading the MFDM performance. In order to verify the performance of the proposed method, the highest PAPR and the average PAPR among them were found by conducting 10 million simulations for each number of phases. As a result, the PAPR was reduced and the performance improved as the number of phases increased. However, when the number of phases increases above a certain level, the increase in operating time is larger than the reduction in the PAPR. Therefore, the SNR was measured by applying stack phase 16 to the 64 × 64 capacitive touch screen panel that was implemented using MATLAB Simulink in this paper. The SNR of the conventional method is 20.01 dB, and the SNR of the P-PTS that utilizes the PTS, which is a technique to reduce the PAPR in communications systems, is 33.38 dB. The SNR of the proposed method is 39.36 dB, which is an advantage of 19.35 dB over the conventional method and 5.98 dB over P-PTS. The PAPR comparison calculation amount that increases exponentially is effectively reduced without changing the structure, and real-time driving is possible. The real-time sensing of 10 multi-touch and the PAPR reduction was verified by outputting it to a monitor by applying the proposed method to the actual 42-inch MFDM CTS. It was confirmed that the proposed method through these performance verifications is capable of high-speed touch sensing even on large touch screens that are 80-inch or higher classes. The future research will reduce the PAPR comparison process through structural modification of the MFDM CTS to further improve the touch sensitivity.

## Figures and Tables

**Table 1:** Environment of the experiment.

Lists	Values
ADC sampling rates	3 MHZ
FFT points	1024
Bandwidth	150 KHz ~ 1 MHz
Number of TX	64
Clock	100 MHz
Number of simulations	100,000,000

**Table 2:** Result of proposed the stack method.

Method					
Stack					
Number of Phases ($n_{p})$	8	16	32	64	128
Average PAPR ($PAPR_{a}$)	7.90	7.33	6.98	6.76	6.65
Maximum PAPR ($PAPR_{m}$)	16.35	13.11	12.05	11.19	10.49
Time (t)	0.0299	0.0504	0.0990	0.1939	0.3830
PAPR-time performance ($PAPR_{a}*\;PAPR_{m}*t)$	3.86	4.84	8.33	14.67	26.72

**Table 3:** Comparison the P-PTS and the stack method.

Method	Simulation Parameters	Computational Complexity (Times)		MaxPAPR	AvgPAPR
P-PTS	$n_{TX\_Total}$ = 288, $n_{TX}$ = 64, $n_{p}$ = 2, $sb_{TX}$ = 8	$n_{p}{}^{sb_{TX}}*\;\frac{n_{TX\_Total}}{sb_{TX}}$	9216	21.90	11.62
Stack (proposed)	$n_{TX\_Total}$ = 288, $n_{TX}$ = 64, $n_{p}$ = 16	$\left(n_{TX}-1\right)*n_{p}$	1008	13.11	7.33

## Data Availability

Not applicable.
